# Local dynamics of topological magnetic defects in the itinerant helimagnet FeGe

**DOI:** 10.1038/ncomms12430

**Published:** 2016-08-18

**Authors:** A. Dussaux, P. Schoenherr, K. Koumpouras, J. Chico, K. Chang, L. Lorenzelli, N. Kanazawa, Y. Tokura, M. Garst, A. Bergman, C. L. Degen, D. Meier

**Affiliations:** 1 Department of Physics, ETH Zürich, Otto Stern Weg 1, Zurich 8093, Switzerland; 2 Department of Materials, ETH Zürich, Vladimir-Prelog-Weg 4, Zurich 8093, Switzerland; 3 Department of Physics and Astronomy, Uppsala University, PO Box 516, Uppsala 75120, Sweden; 4 Department of Applied Physics, University of Tokyo, Tokyo 113-8656, Japan; 5 RIKEN Center for Emergent Matter Science (CEMS), Wako 351-0198, Japan; 6 Institute for Theoretical Physics, Universität zu Köln, Köln D-50937, Germany; 7 Department of Materials Science and Engineering, Norwegian University of Science and Technology, Trondheim 7491, Norway

## Abstract

Chiral magnetic interactions induce complex spin textures including helical and conical spin spirals, as well as particle-like objects such as magnetic skyrmions and merons. These spin textures are the basis for innovative device paradigms and give rise to exotic topological phenomena, thus being of interest for both applied and fundamental sciences. Present key questions address the dynamics of the spin system and emergent topological defects. Here we analyse the micromagnetic dynamics in the helimagnetic phase of FeGe. By combining magnetic force microscopy, single-spin magnetometry and Landau–Lifschitz–Gilbert simulations we show that the nanoscale dynamics are governed by the depinning and subsequent motion of magnetic edge dislocations. The motion of these topologically stable objects triggers perturbations that can propagate over mesoscopic length scales. The observation of stochastic instabilities in the micromagnetic structure provides insight to the spatio-temporal dynamics of itinerant helimagnets and topological defects, and discloses open challenges regarding their technological usage.

## Abstract (web summary)

Topological defects may strongly influence the evolution of a materials' micromagnetic structure whilst their manipulation forms the basis for emerging technological concepts. Here, the authors study the depinning and motion of magnetic edge dislocations in the domain structure of helimagnetic FeGe.

## Graphical abstract

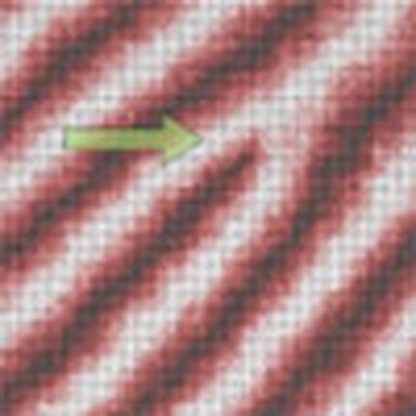

Intriguing states of magnetism^1^ arise in transition-metal silicides and germanides of the B20-type such as MnSi^2^,^3^, Fe_1−*x*_Co_*x*_Si^4^,^5^,^6^, and FeGe^7^,^8^,^9^. The competition of ferromagnetic exchange, Dzyaloshinskii–Moriya (DM) interaction, and magnetic anisotropy leads to a variety of complex magnetic phases with spins forming helical or conical spirals, as well as long-range-ordered lattices of magnetic whirls^10^. These spin structures are appealing as they give rise to anomalous transport properties^11^,^12^, exotic vortex domain walls^13^ and unusual dynamic spin-wave phenomena^14^,^15^,^16^. Of particular interest is the emergence of topologically protected spin states, that is, stable magnetic configurations that cannot be generated or destroyed by a continuous transformation of the spin system^17^. These topological defects are explicitly robust and may serve as functional objects in future spintronics devices^18^,^19^. At present, however, we are only at the verge of grasping the technological potential of topological spin states^20^,^21^ and their complex nanoscale physics is still largely unexplored.

During the last years, research activities in the field mainly focused on topologically protected magnetic whirls called skyrmions. Skyrmions arise in various B20 materials under magnetic fields and represent particle-like entities that can be moved^12^, written and erased on demand^22^. Although it is known that the formation of skyrmions is facilitated by superior topological defects that develop in the helimagnetic ground state^23^, little attention has been paid to the latter ones. In the helimagnetic phase topological defects arise, for example, in the form of magnetic edge dislocations^24^,^25^. Analogous to edge dislocations in crystals and nematics, these magnetic edge dislocations naturally develop where helical spin textures of unequal phase meet, compensating for the local mismatch. At the bulk level, such line-like topological defects are often neglected as they affect only a small fraction of the volume. At the nanoscale, however, the defects and their dynamics become crucial as they can lead to significant perturbations in the electronic structure in itinerant helimagnets. Thus, due to the close relation to the formation of the skyrmion phase and their general significance for the research on topological states, a detailed knowledge about the dynamics of topological defects in the helimagnetic state is highly desirable.

The probing of intrinsic micromagnetic instabilities at the nanoscale in a non-invasive way is a well-known challenge. Conventional microscopy methods, such as Lorentz transmission electron microscopy, magnetic force microscopy (MFM)^26^ and scanning tunnelling microscopy^27^, make either use of an electron beam or a magnetic probe tip and can themselves influence the behaviour of the spin structure. As a consequence, it is difficult to unambiguously separate between intrinsic and extrinsic, probe-induced dynamical effects. Nitrogen vacancy (NV) centre-based magnetometry^28^,^29^ is a rather new experimental method that, in principle, is capable of providing the desired information. This technique has already been used successfully to study, for example, vortices^30^, domain walls^31^,^32^ and spin wave excitations^33^,^34^ in ferromagnets, but it has never been used to probe (helical) antiferromagnetic spin arrangements and rarely been applied under cryogenic conditions^35^,^36^.

In this article, we study emergent micromagnetic dynamics in the helimagnetic phase of FeGe based on MFM, NV centre magnetometry, and Landau–Lifschitz–Gilbert (LLG) simulations. The MFM measurements reveal temperature-driven local changes in the magnetic domain structure, as well as jump-like collective movements of the helical spin texture that propagate over mesoscopic length scales. The collective movements are driven by the depinning and subsequent motion of topological magnetic edge dislocations by which the system relaxes its magnetic structure. Single-spin magnetometry experiments with NV centres, immobilized on the FeGe surface, show that these dynamics are intrinsic and highlight their stochastic nature. Coarse-grained LLG simulations are applied to analyse the microscopic magnetization dynamics. The simulations demonstrate that the movement and annihilation of topological defects plays a key role for the self-organization of the spin structure and the development of a long-range-ordered helimagnetic ground state.

## Results

### Helimagnetism in FeGe probed by magnetic force microscopy

**Figure 1:**
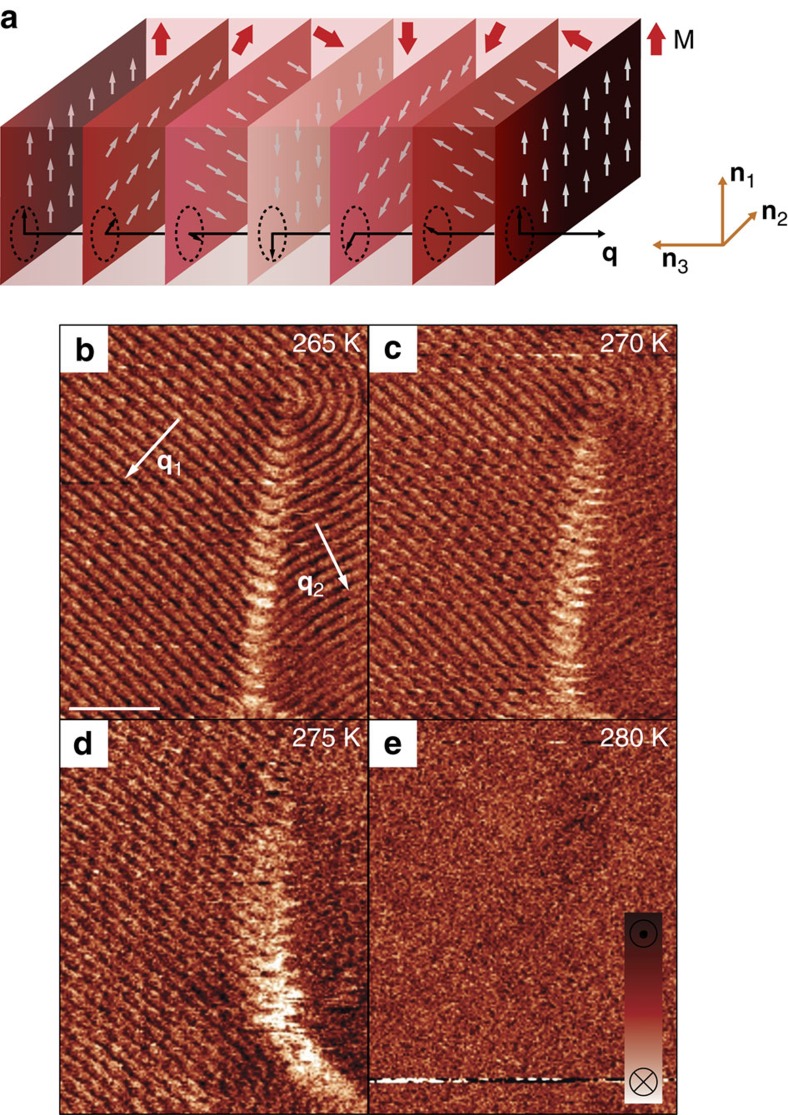
Temperature dependence of the helimagnetic domain structure in FeGe. (**a**) Schematic of the helimagnetic spin order and wave vector **q**. Colour-coded planes indicate wave fronts of the magnetization M that are defined by uniformly oriented spins. (**b**) MFM image in the helimagnetic state. **q**_1_ and **q**_2_ indicate two magnetic domains. Scale bar, 500 nm. (**c**,**d**) With increasing temperature, local variations are observed in the domain structure while the period of the helimagnetic order within domains is unaffected. (**e**) The magnetic contrast vanishes when approaching *T*_N_.

For our studies on the dynamics of topological magnetic defects, we choose cubic FeGe^37^ as it exhibits helimagnetic order near room temperature with *T*_N_≈280 K and because its phase diagram is well-characterized^38^. Single crystals of FeGe were grown by the chemical vapour transport method^39^ as explained in the Methods section. The helical axis of the spin system in FeGe is described by a wave vector **q**, which first points along the crystallographic 〈001〉 direction (Fig. 1a), changing to the 〈111〉 direction below 211 K on cooling^8^. We begin our discussion with the spatially resolved MFM measurements shown in Fig. 1b–e. The MFM data are collected in two-path mode, recording first the topography in semi-contact and then the magnetic response with a fixed tip-surface distance of about 30 nm (a representative topography image is shown in Supplementary Fig. 1; see Methods section for further technical details). After cooling the sample to 265 K, alternating bright and dark lines are clearly visible, indicating a periodic magnetic structure. To relate the MFM data to the microscopic spin arrangement, we calculate the magnetic stray field for helimagnetic order with periodicity *λ* and a constant magnetization amplitude |**M**|=*M* (see Fig. 1a for a schematic illustration of the helical spin structure). The magnetic structure can be described as


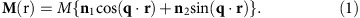


Here the **n**_*i*_ (*i*=1,2,3) define a set of orthonormal unit vectors and **q**=**n**_3_2*π*/*λ*. For a sample with **q** lying in the surface plane and *z*||**n**_1_ being the probe distance above the sample surface, the spin helix described by equation (1) leads to a magnetic stray field


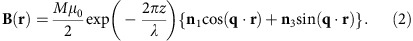


Equation (2) reflects that the periodicity of the stray field is equal to the periodicity *λ* of the spin helix, and that the stray field exponentially drops with vertical decay length *λ*/(2*π*)≈11 nm. Note that while the periodicities of **M**(**r**) and **B**(**r**) are the same, their rotation axes are orthogonal and defined by **n**_3_ and **n**_2_, respectively. The calculated magnetic stray field is in qualitative agreement with the MFM data in Fig. 1 and we find *λ*=70±5 nm, which is consistent with neutron scattering data^8^. The measurement in Fig. 1b further reveals micrometer-sized magnetic domains with different orientation of the wave vector **q**, that are separated by a so-called vortex-free domain wall as detailed in ref. 13.

To investigate the stability of the helimagnetic order, we perform additional MFM scans at elevated temperature as illustrated in Fig. 1c–e. A comparison of the MFM images shows that the magnetic stray field associated with the structure of the domain wall between the **q**_1_- and **q**_2_-domain slightly varies with temperature, revealing a change in the length and orientation of the wall. The periodicity of the spin helix within the domains, by contrast, is robust against the temperature-driven variation in the domain pattern within the time-frame capture by the scan.

### Micromagnetic relaxation dynamics

**Figure 2:**
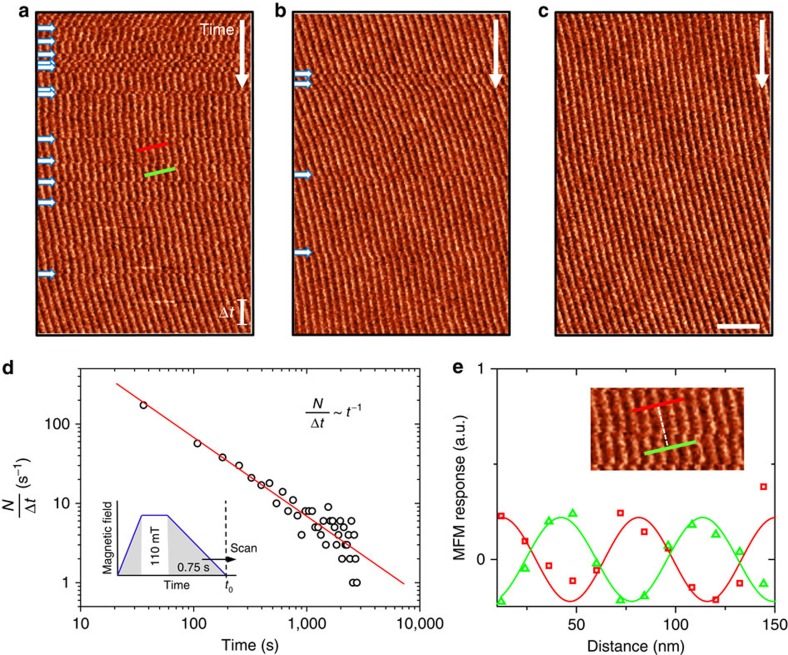
Dynamical phase jumps and relaxation behaviour of helimagnetic order. (**a**–**c**) Representative MFM image series gained at the same sample posititon at *H*=0 T after applying a magnetic field of 110 mT as sketched in the inset to **d**. Scan lines are recorded from top to bottom. The data reflect the emergence of stochastic collective jumps in the spin system, indicated by white arrows, which get less frequent as the scan progresses. The scale bar in **a** corresponds to a time frame of Δ*t=*70 s. Scale bar in **c**, 400 nm. (**d**) Time-dependence of the rate of phase jumps, *N*/Δ*t*, extracted from over 40 MFM image series as seen in **a**–**c** (see text for details). The graph reflects a relaxation that follows a power law with *N*/Δ*t∝t*^−1^. (**e**) Evaluation of the change in period for the jump-like event shown in the inset (zoom-in to the area marked in **a**).

Occasionally, we observe jump-like collective movements in the helical spin structure while imaging. One may speculate that these movements are triggered by tip–sample interactions, caused by scanning with a magnetic MFM probe tip. The jumps, however, are especially visible after the spin system has been disturbed by a magnetic field or a change in temperature, and eventually vanish after multiple scans. This behaviour discards tip-induced effects as the only source of the jump-like movements and points towards an intrinsic phenomenon. Figure 2a–c shows an MFM image series gained in the helical state after driving the system into the magnetic field-aligned phase, as sketched in the inset of Fig. 2d. A systematic analysis of more than 40 time-dependent MFM experiments (performed at different sample positions) shows that the number of jumps *N* per time interval Δ*t*=70 s follows a power law as known from slow relaxation processes (Fig. 2d)^40^; we find 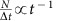. Interestingly, there is always a phase change associated with the individual jumps (Fig. 2e), often around 180°.

**Figure 3:**
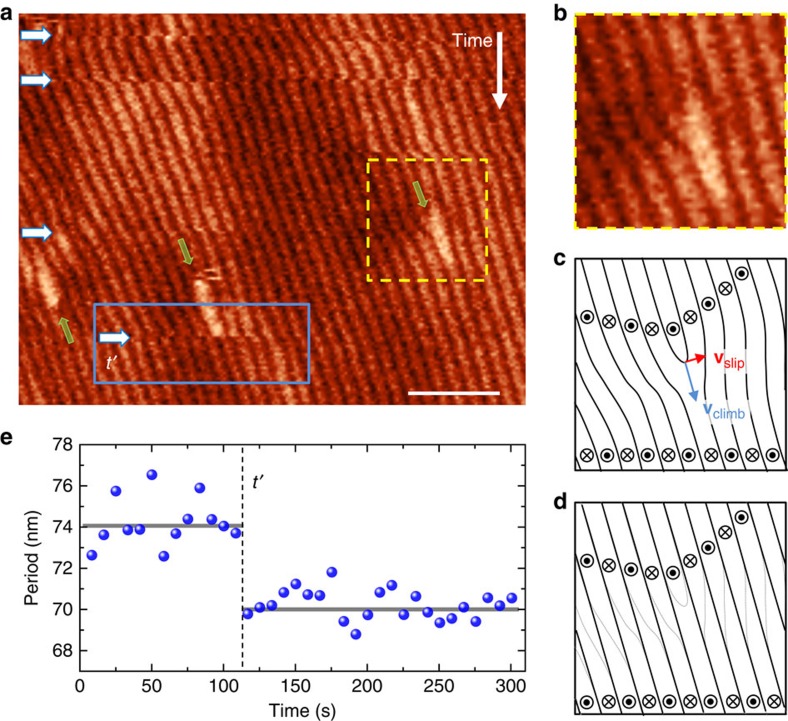
Static and mobile magnetic edge dislocations. (**a**) MFM image of the helimagnetic structure at 266 K displaying several topological defects (magnetic edge dislocations; indicated by green arrows) and stochastic magnetization jumps (white arrows). The orientation of the green arrows indicates that emergent magnetic edge dislocations have different relative orientations. An extra half-plane of spins, here with out-of-plane orientation (dark), occurs either below or above the defect, which is conventionally expressed by refering to the defect as negative or positive edge dislocation, respectively. Scan lines are recorded from top to bottom. Scale bar, 500 nm. (**b**) Zoom-in to the area highlighted by the yellow dashed box in **a**, presenting a magnetic edge dislocation. (**c**) Schematic illustration of the edge dislocation seen in **b**. Black symbols indicate the direction of the out-of-plane component of the magnetic stray field, and arrows reflect the directions for slip and climb motions of the defect. (**d**) Illustration of the structure in **c** after the defect climbed out of the field of view, yielding a 180° phase jump in the lower region of the sketch. (**e**) Evolution of the local magnetic period in the wake of the defect as function of time, evaluated for the blue solid box in **a**. At *t*′ the local mean period abruptly changes by about 4 nm (see white arrow in **a**).

Figure 3 presents a possible relaxation mechanism, driven by the dynamics of magnetic defects, causing such collective jump-like movements. The image in Fig. 3a is recorded at *T*=266 K in a surface area with magnetic defects (marked by green arrows). These defects exhibit a locally enhanced magnetic stray field, leading to a brighter contrast level compared with the surrounding periodic spin structure. A closer inspection of the defects identifies them as magnetic edge dislocations as shown by the zoom-in and the corresponding sketch in Fig. 3b,c, respectively. Edge dislocations are line-like topological defects that, in the present case, allow the system to compensate for mismatches in the periodicity of its spin structure^24^,^41^. Analogous to their crystallographic counterpart, two types of magnetic edge dislocations can be distinguished (positive or negative), depending on the relative position of the respective extra half-plane of spins as detailed in the caption of Fig. 3a. The observation of these magnetic edge dislocations in bulk FeGe complements earlier data obtained by Lorentz transmission electron microscopy on thin platelets of FeGe^42^, Fe_1−*x*_Co_*x*_Si^24^,^25^ and BaFe_12−*x−y*_Sc_*x*_Mg_*y*_O_19_ (ref. 23).

The magnetization dynamics presented before in Fig. 2 can be understood by assuming that magnetic edge dislocations spontaneously unpin and climb along the helical plane (that is, perpendicular to **q**, Fig. 3c,d). An example of such a spontaneous unpinning is shown in Fig. 3e. Here a jump of the spin system is captured at time 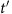 that can be connected to the magnetic edge dislocation, which, at time 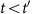, was situated about 350 nm above the solid blue box in Fig. 3a. The movement of this dislocation locally relaxes the initially stretched magnetic period (≈74 nm) to its equilibrium value of 70 nm, removing the tension that was associated with the previously pinned dislocation (Fig. 3e). The relaxation of the tension is thus achieved by reducing the local density *n* of edge dislocations so that we conclude *n*∝*t*^−1^.

The climbing of magnetic edge dislocations can also explain the tendency of the system to perform dynamical phase jumps of about 180° that can extend over many micrometres (see Fig. 3c,d for an illustration). The associated climb velocity is expected to be fast; a lower limit for 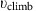 can be derived from Fig. 3a based on the distance the defect travelled (Δ*d*≳350 nm) and the time difference between two consecutive scan lines (Δ*t*=8 s). We find 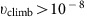 m s^−1^, which would be comparable to slowly moving structural dislocations^43^,^44^; however, our 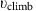 is a lower bound and the actual velocity may be much faster. In case of a defect-free magnetic environment we usually observe phase shifts to propagate across the entire field of view (≳10 μm). Such a long-distance propagation is possible because of the incommensurability of the spin structure. Due to the incommensurability the free energy is independent of the helical phase and phase shifts cost no energy^45^. Thus, once launched, the energy gain associated with the local relaxation of the spin system can readily sustain the defect movement and the phase shift in its wake. Only the presence of pinned magnetic or structural defects, as well as domain walls, eventually halts the free propagation and confine the affected area.

### Accessing helimagnetism by NV centre-based magnetometry

**Figure 4:**
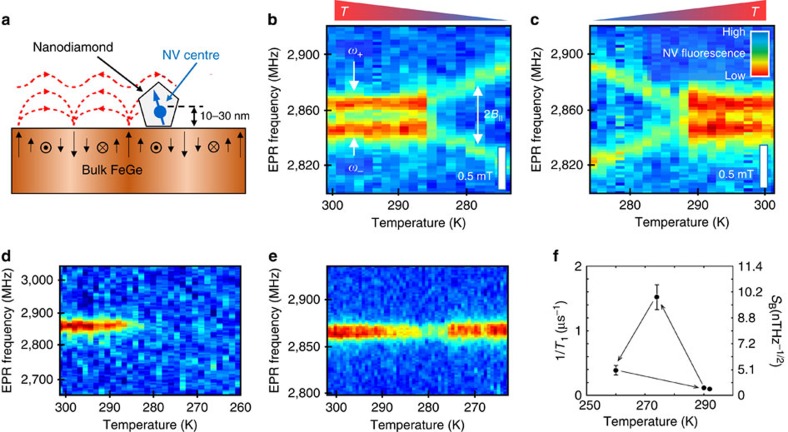
Dynamical variations in the local magnetic structure observed by single-spin magnetometry. (**a**) Measurement schematic: nanodiamond containing a single NV centre is immobilized on the FeGe surface. The local magnetic stray field (dashed red lines) induces Zeeman shifts to the NV centre's electronic spin transitions (*m*_*s*_=0↔*m*_*s*_=±1) that are measured using optically detected EPR. Black arrows indicate the helical spin texture of FeGe. (**b**,**c**) Optically detected EPR spectrum during cool-down and warm-up, respectively, revealing the paramagnetic-to-helimagnetic phase transition at *T*_N_≈286 K. Colour coding reflects normalized fluorescence intensity. (**d**,**e**) EPR signal of two additional NV centres, representing the two other characteristic types of traces that were observed. (**f**) *T*_1_ measured above, at, and below *T*_N_ for an NV centre that showed a trace similar to **e**. Arrows indicate order of measurement. The scale on the right side provides the magnetic noise spectral density at the EPR frequency (∼2.9 GHz) calculated as *S*_*B*_=2/(γ^2^*T*_1_), where γ is the electron gyromagnetic ratio. *T*_1_ values were measured using the protocol of ref. 56.

To verify that the magnetization dynamics are intrinsic to FeGe and not triggered by the stray field of the MFM probe tip, we conduct a non-invasive magnetometry measurement with single NV centres in diamond^28^,^29^,^31^,^33^ (Methods section). As illustrated in Fig. 4a, we disperse diamond nanocrystals on the FeGe surface such that the NV centres are sufficiently close (∼10–30 nm) to pick up the local helimagnetic stray field. Since the NV centres are immobilized on the surface, they can directly record any relative movement of the spin texture with respect to the underlying crystalline lattice of FeGe. Magnetometry measurements are performed by monitoring the two electron paramagnetic resonance (EPR) transitions of the NV electronic spin using optical detection^28^. The difference between the two EPR frequencies, denoted by *ω*_+_ and *ω*_−_ in Fig. 4b, represents a Zeeman splitting that is proportional to the local magnetic stray field,


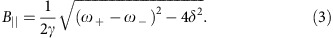


Here *γ*=2*π* × 28 GHz T^−1^ is the electron gyromagnetic ratio, *δ* is an additional splitting caused by strain in the nanocrystal, and *B*_||_ is the component of **B**(**r**) along the NV spin direction^46^. At the same time, the sum of the two EPR frequencies can be used to monitor the local temperature *T* via the (temperature-dependent) zero-field splitting parameter *D* which, for small magnetic fields, can be expressed as


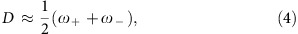


with *D*≈2,867 MHz–0.074 MHz × (*T*−293 K) K^−1^ (ref. 47). An EPR datapoint thus provides a simultaneous measurement of the local magnetic field and the local temperature.

Since the technique of NV magnetometry is relatively recent^28^ and has never been applied to the study of antiferromagnetic order, and rarely at low temperature^35^,^36^, we first demonstrate that the method is sensitive to the onset of helimagnetism. Figure 4b,c presents temperature scans across the phase transition. Below *T*_N_=286±3 K a pronounced Zeeman splitting is observed in the EPR signal, corresponding to an increase of *B*_||_ from 0 to 1.2 mT. The EPR splitting reversibly vanishes when returning to above *T*_N_. The measurement thus clearly shows the sensitivity of NV magnetometry to the helimagnetic order. The value of *T*_N_ found here is somewhat higher than expected from the MFM data (Fig. 1e) and literature values^8^, most likely due to the limited accuracy of the absolute temperature calibration of *D*.

Not all NV centres showed the response displayed in Fig. 4b,c, as the placement of nanodiamonds is stochastic and the vertical distance to the FeGe surface varies from NV centre to NV centre. Figure 4d,e shows two additional characteristic behaviours observed with other NV centres. In Fig. 4d, the EPR signal disappears entirely below *T*_N_, presumably due to fluorescence quenching by either a strong off-axis magnetic field^48^ or rapid nuclear spin relaxation. In Fig. 4e, no Zeeman splitting is observed in the helimagnetic phase, because the NV is relatively distant and the stray field is small (see Supplementary Fig. 2 for further details). Interestingly, almost all traces show a pronounced reduction of the fluorescence contrast at *T*_N_. Figure 4f shows that this dip in fluorescence is accompanied by a sharp reduction in the spin relaxation time *T*_1_ (see also Supplementary Note 1). Since short *T*_1_ times are indicative of magnetic noise at the EPR transition frequency, the fluorescence reduction is likely caused by increased magnetic fluctuations at the phase transition. Such fluctuations are expected from magnetic instabilities at the local scale that peak around *T*_N_^49^.

### Thermally driven magnetization dynamics

**Figure 5:**
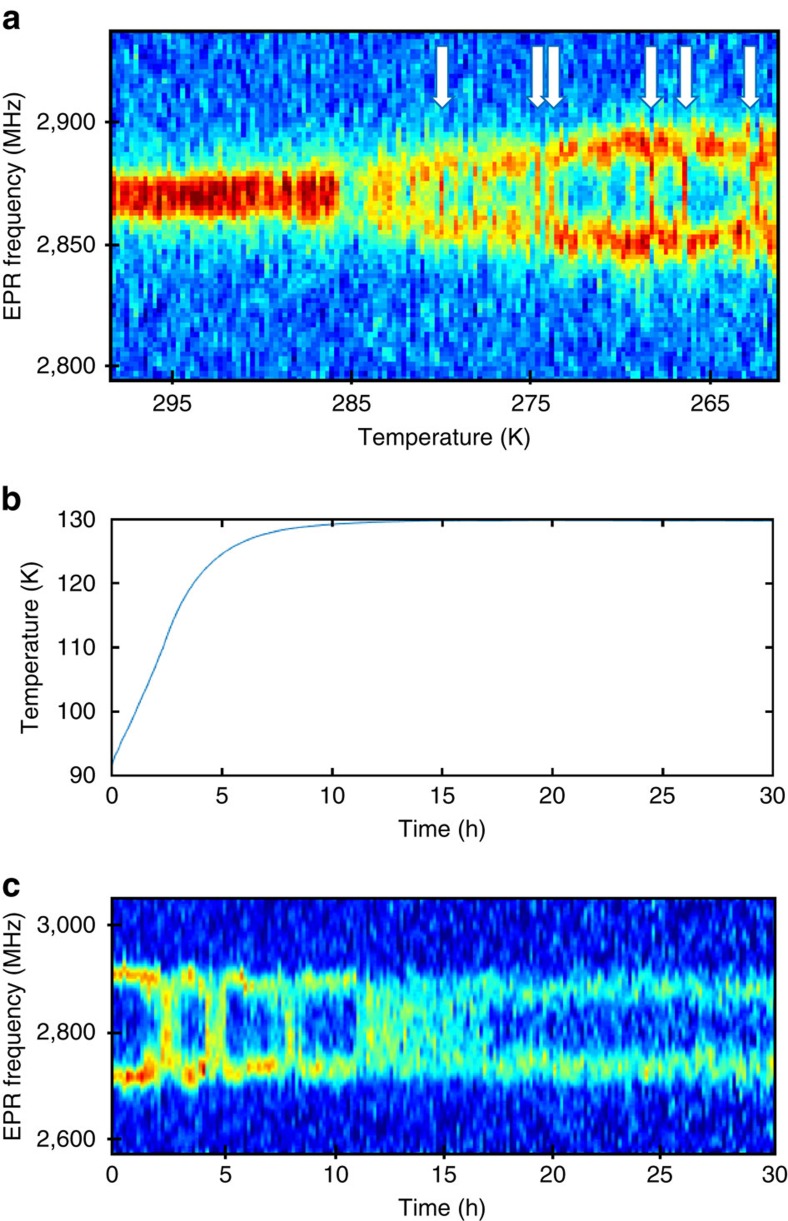
Transient breakdowns in the optically detected EPR signal. (**a**) EPR signal of an NV centre showing a splitting below *T*_N_. The transient breakdowns in the EPR signal below *T*_N_ indicate a sudden change of the local magnetization probed by the NV center (white arrows). (**b**,**c**) Temperature evolution and corresponding EPR signal. Transient breakdowns in the EPR signal are detected as long as the temperature changes and vanish completely after the temperature is stabilized.

After discussing the NV centre response to the onset of helimagnetism, we now turn to the detection of dynamical magnetic variations. Figure 5a presents the optically detected EPR signal of a different NV centre, recorded with decreasing temperature. In agreement with Fig. 4b, a pronounced Zeeman splitting is visible below *T*_N_. In addition, we find that the splitting transiently breaks down while cooling as indicated by the white arrows in Fig. 5a. Note that the maximum excursion of the Zeeman splitting varies only slowly with temperature, despite the many breakdowns, and assumes a roughly constant value below *T*∼275 K. This behaviour indicates that the breakdowns are associated with sudden changes in the orientation of the local magnetization, that is, |**B**| remains constant. These findings are consistent with the spin system's tendency towards phase jumps of about 180° obtained by MFM. Supplementary Fig. 3 shows that transients are also observed at temperatures far below *T*_N_. Opposite to the magnetically stimulated phase jumps observed by MFM (Fig. 3), however, the phase jumps observed with NV centres are caused by a change in temperature. The latter is reflected by Fig. 5b,c, which confirms that the breakdowns are absent when the temperature is held constant for a long time.

### Micromagnetic simulation of moving edge dislocations

To develop a microscopic model of the captured dynamics we perform simulations based on the LLG equation. We model the helimagnetism of FeGe with Heisenberg and DM exchange interactions obtained from electronic structure calculations as input parameters (Methods section). This model yields a magnetic ground state with a perfect helical spin arrangement of period *λ*≈100 nm and *T*_N_=240 K, which is in fair agreement with the experimental observations. For *T*>0 K magnetic fluctuations occur at the atomic scale and locally disturb the helimagnetic order (Supplementary Fig. 4). Such magnetic excitations increase towards *T*_N_ and ultimately destroy the magnetic order, consistent with the MFM data shown in Fig. 1 and the NV data in Fig. 4f.

**Figure 6:**
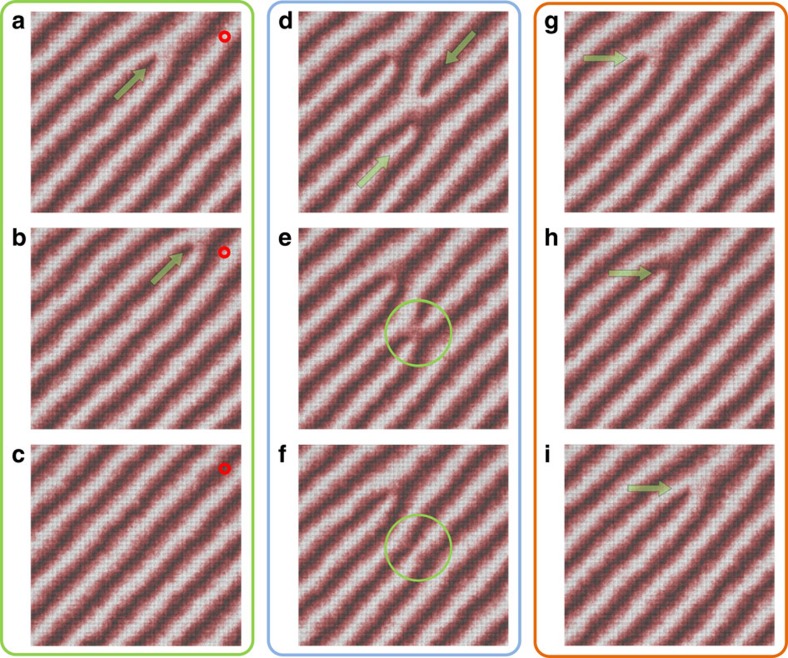
LLG simulations of emergent micromagnetic dynamics in the helimagnetic state. (**a**–**c**) Simulations performed at *T*=0.5̇*T*_N_ reveal that magnetic edge dislocations (green arrow) can climb through the helimagnetic structure. The movement locally relaxes the spin structure and induces a phase jump of 180° as seen, for example, at the position marked by the red dot. (**d**–**f**) Pair annihilation occurs when positive and negative edge dislocations meet. (**g**–**i**) In addition to the climbing in **a** magnetic edge dislocations can perform slip motions (*T*=0.5̇*T*_N_).

In addition to these local fluctuations, long-range magnetic excitations arise close to *T*_N_ that break the helimagnetic structure and naturally lead to the formation of positive and negative magnetic edge dislocations as introduced before (see also Fig. 3a). On thermal quenching these edge dislocations remain quasi-stable as presented in Fig. 6. The quasi-stability is expected due to the topological nature of these magnetic excitations. The simulations further highlight that the magnetic edge dislocations are quite mobile. Figure 6a–c shows they can easily climb through the helical spin structure (**v**_climb_⊥**q**). The climbing motion relaxes the local magnetic order and triggers phase shifts in the helimagnetic structure (see red circle in Fig. 6a–c), analogous to the illustration in Fig. 3c,d, which corroborates the above interpretation of our experimental data. Whenever positive and negative edge dislocations meet, they annihilate which further lowers the magnetic energy (Fig. 6d–f).

Interestingly, the micromagnetic simulations reveal that the magnetic edge dislocations can also move parallel to the wave vector **q**, that is, with **v**_slip_||**q** as shown in Fig. 6g–i. The emergence of slip motions is surprising because slipping involves the destruction and creation of topolicial defects, but does not lead to an immediate relaxation of the spin system. Altogether, the micromagnetic calculations show that three types of magnetic defect dynamics, namely climbing, slipping, and pair annihilation, emerge at finite temperature in the helimagnetic phase. With this, the LLG simulations demonstrate a striking analogy between the dynamics of magnetic edge dislocations in FeGe and topological defects in crystals and nematics.

## Discussion

In summary, we have investigated the dynamics of topological magnetic defects in FeGe. By combining MFM, single-spin magnetometry with NV centres, and LLG simulations we demonstrated that mobile magnetic edge dislocations play a key factor in the development of the helimagnetic ground state. Their movements help the system to order and reduce its free energy, but they also lead to stochastic perturbations which can propagate over microscopic distances and which may explain the emergence of spontaneous magnetic instabilities in helical magnets^50^. Such perturbations increase the noise level and need to be controlled adequately in envisaged device applications. We were able to generate micromagnetic instabilities both by a magnetic field ramp and small changes of temperature. Analogous to magnetic monopoles^6^, which are involved in the formation of skyrmion states, the magnetic edge dislocations discussed in our work are able to move through the helimagnetic spin texture. The obtained defect dynamics point towards fundamental similarities in the transportation of topological defects in electronic spin liquids and nematics, and reveal an intriguing connection between the micromagnetic dynamics in itinerant helimagnets and the self-organization of large-scale dynamic structures.

## Methods

### Samples

Single crystals of FeGe were grown by the chemical vapour transport method^39^. Powder of FeGe with B35-type crystal structure was placed with I_2_ (20 mg) in an evacuated quartz tube. The tube was mounted in a three-zone furnace and heated for 1 month under a thermal gradient, that is, 560 °C at the end of the cylinder where the powder sample was placed and 500 °C at the other end of the cylinder. This led to the growth of B20-type FeGe single crystals at the lower temperature side of the cylinder. The B20 crystal structure was confirmed by Laue diffraction and oriented specimens with a thickness of about 500 μm and a lateral extension of 1 × 1 mm were achieved for microscopy experiments. Flat surfaces were achieved by chemo-mechanical polishing using silica slurry, yielding a surface roughness below 1 nm.

### Magnetic force microscopy

All MFM data were recorded with a commercial magnetic tip (Nanosensors, PPP-MFMR, resolution <50 nm) in two-pass mode, that is, MFM imaging was perform after recording the surface topography in semi-contact with a tip-surface distance of 30 nm in the second scan. The scanning probe system was operated at the resonance frequency of the magnetic tips, which was around 75–77 kHz. Optimal magnetic imaging was achieved with an image resolution of 10–15 nm per measuring point and a scan speed of 2–3.5 μm s^−1^. To access the helimagnetic phase of FeGe, samples were cooled using a home-built low-temperature holder based on a water-cooled three-stage peltier element^51^. The holder was implemented into a commercially available scanning probe microscope (NT-MDT). Low flow rates allowed for minimizing vibrations due to the water cooling. To prevent ice from building-up on the samples surface, all measurements were performed in a dry nitrogen environment (humidity below 1%).

### NV centre-based magnetometry

Single-spin magnetometry experiments were carried out on a home-built confocal microscope housed in a dry optical cryostat (Montana Instruments Cryostation). NV centres were illuminated using green 532-nm laser light and the fluorescence was detected through a 630–800-nm bandpass filter using a single photon counter module (Excelitas SPCM-AQRH). Microwaves were generated using a synthesizer with adjustable frequency and power level (Quicksyn Phasematrix), amplified, and directed through a thin wire that passed in close proximity (∼100 μm) of the NV centre. Optically detected EPR spectra were taken by stepping the microwave frequency through resonance and recording the photon counts for each frequency. Nanodiamonds with a nominal diameter of 25 nm and typically ∼1 NV per crystal (DiaScence, Van Moppes) were dispersed at low density on the FeGe surface such that single NV centres could be optically resolved. The FeGe sample was mounted on a sapphire holder and thermally anchored on an OFHC copper sample stage that was cooled via a cold finger. To avoid effects of local heating, the microwave wire was not allowed to touch the FeGe sample and low laser powers (≈80 μW) were used for the optical readout. The temperature of the sample was simultaneously monitored via the temperature-dependent EPR response of the NV centre, and by a conventional thermometer attached to the sapphire holder. We found that while local heating could be induced by high laser and microwave powers, it could be avoided by reducing the power level. From 21 NV centres whose response was analysed across the phase transition, 5 showed no signs of helimagnetism, 6 showed a splitting similar to Fig. 4b, 6 showed suppressed contrast below *T*_N_ as in Fig. 4d, and 4 showed suppressed contrast only around *T*_N_ as in Fig. 4e.

### Micromagnetic simulations

We applied a multiscale approach to model the helimagnetism in FeGe. First, we obtained the electronic structure and magnetic properties by performing first principles calculations of FeGe in the B20 structure with a lattice parameter of 4.7 Å (ref. 8). The calculations were performed via the fully relativistic KKR method as implemented in the SPR-KKR package^52^. The shape of the potential was approximated via the Atomic Sphere Approximation (ASA) and the exchange correlation potential was treated via the Local Spin Density Approximation (LSDA) as parametrized by Vosko, Wilk and Nusair (VWN)^53^. Using the same method, both Heisenberg and DM exchange interactions were calculated^54^. These interactions define the spin Hamiltonian which served as the basis for the numerical simulations, where we used the Uppsala Atomistic Spin Dynamics package^55^ both for LLG and Monte Carlo simulations. The spin Hamiltonian was defined for atomic spins, but since the length scale for the helical spin state in FeGe is long compared with the atomic length scale, we performed coarse-grained simulations in addition to the atomistic simulations. In our coarse-graining scheme we still simulated discrete magnetic moments, but each discrete moment then represented the magnetization of a larger volume of the sample, from 1 × 1 × 1 to 5 × 5 × 5 nm. The interactions between the volume elements were then renormalized so that the effective exchange interactions corresponded to the same spin-wave stiffness and DM stiffness as in the atomistic situation. Coarse-graining the system like this gave a good description of long-wavelength fluctuations.

### Data availability

The data that support the findings of this study are available from the corresponding author on request.

## Additional information

**How to cite this article:** Dussaux, A. *et al*. Local dynamics of topological magnetic defects in the itinerant helimagnet FeGe. *Nat. Commun.* 7:12430 doi: 10.1038/ncomms12430 (2016).

## Supplementary Material

Supplementary Information Supplementary Figures 1-4, Supplementary Note 1 and Supplementary References.
