# Supplementary material for: Local dynamics of topological magnetic defects in the itinerant helimagnet FeGe
Source: Nat Commun. 2016 Aug 18;7:12430. doi: 10.1038/ncomms12430 (PMC4992142; doi:10.1038/ncomms12430)
Supplement: Supplementary Information — Supplementary Figures 1-4, Supplementary Note 1 and Supplementary References. [file ncomms12430-s1.pdf]

Supplementary Figure 1

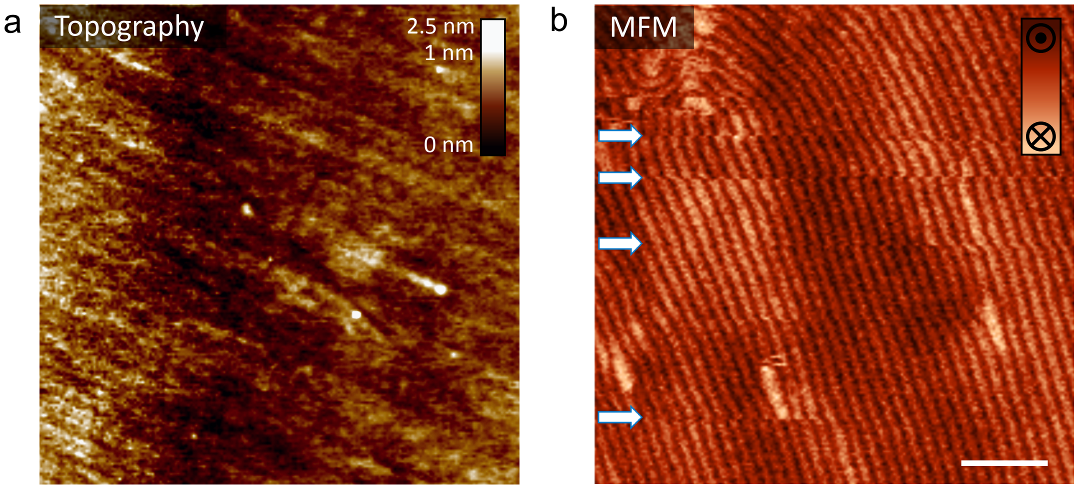

Topography and corresponding MFM image gained in a two-path measurement. a, Representative topography image gained on FeGe polished with silica slurry, reflecting a surface roughness below 1 nm. **b**, Magnetic pattern imaged together with the topography scan in **a**. A comparison of the two data sets shows that the spontaneous jumps discussed in the main text occur only in the magnetic structure, without any indication of related line-to-line discontinuities in the topography scan. Scale bar, 500 nm.

Supplementary Figure 2

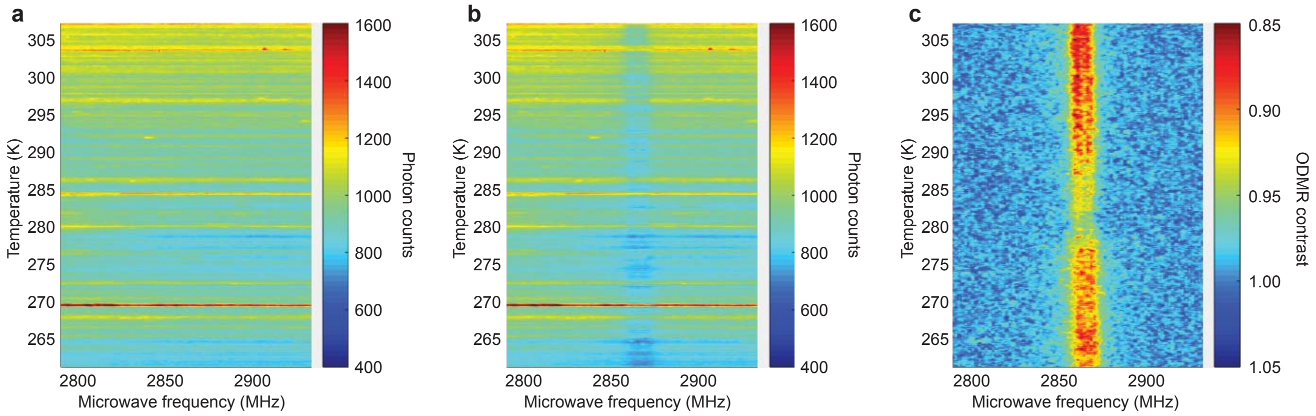

Example ODMR dataset, corresponding to Fig. 4(e) in the main manuscript. **a**, Reference photon counts measured when microwaves were off. **b**, Signal photon counts measured when microwaves were on. **c**, Reported ODMR contrast, obtained by dividing signal counts by reference counts. Clearly visible, the large intensity fluctuations seen in (a) and (b) are efficiently rejected. The fluctuations are mainly due to thermal drifts and automated repositioning of the optics.

Supplementary Figure 3

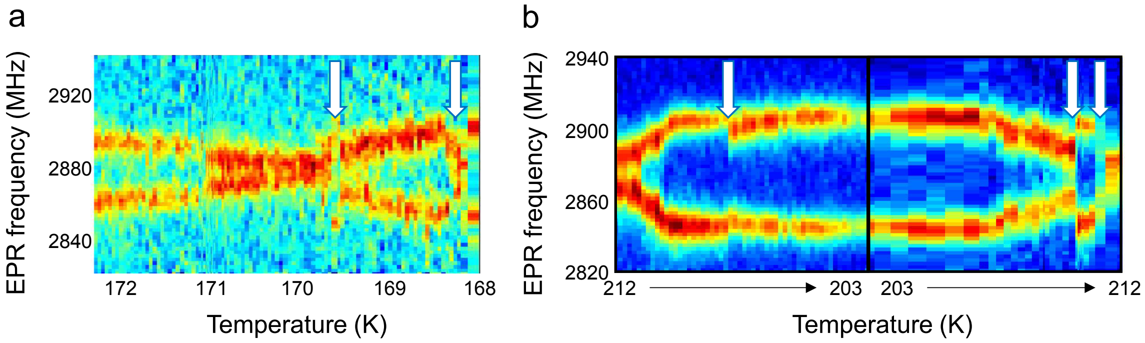

Quasi-reversible local dynamics probed by single-spin magnetometry. a, Besides the observed transient breakdowns in the EPR response (indicated by white arrows), a qualitatively different, second type of anomaly emerges in the response of specific NV centers as function of temperature. The Zeeman splitting continuously decreases with decreasing temperature between 172.5 K and 170 K and then reemerges with further decreasing temperature. The variation in the Zeeman splitting reflects a change in either the magnitude or orientation of the local magnetic stray field, with an amplitude of about 0.8 mT. Note that the gradual variation in the Zeeman splitting does not reflect a magnetic phase transition. This is evident from the FeGe phase diagram and the fact that the temperature at which the EPR signal vanishes is specific to the evaluated NV center. As the temperature is lowered, the crystal lattice contracts, leading to slow changes in the local periodicity of the weakly pinned spin helix. **b**, The slow temperature-dependent Zeeman splitting is reversible during the reversible compression of the spin helix when the temperature is ramped between 213 K and 203 K and coexists with the sudden jumps discussed in the main text.

Supplementary Figure 4

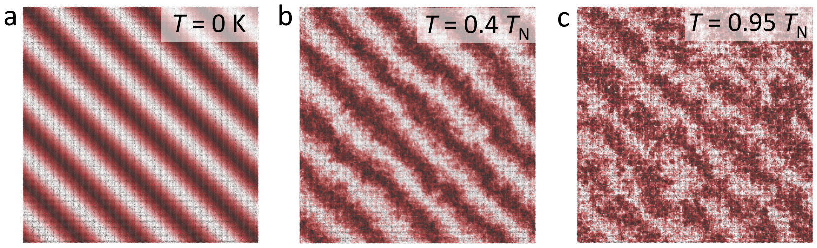

Simulated temperature-dependent helimagnetism in FeGe. a, In the magnetic ground state ($T = 0$ K), FeGe displays a perfectly order helimagnetic spin arrangement, leading to a stripe-like patter when calculating the magnetic out-of-plane component (dark = $+M$, bright = $-M$). **b**, At finite temperature ($T = 0.4 \cdot T_{\mathrm{N}}$) fluctuations arise at the atomic scale that locally perturb the helimagnetic order. **c**, Toward T_{N} ($T = 0.95 \cdot T_{\mathrm{N}}$) the local fluctuations increase and eventually destroy the long-range magnetic order, leading to a paramagnetic state for $T > T_{\mathrm{N}}$.

Supplementary Note 1: Protocol used for measuring T_1 relaxation times

Spin relaxation times T_1 were measured using the procedure presented in Supplementary Reference 1. To record a T_1 curve, the NV center was pumped into the $m_S = 0$ state by a short laser pulse, followed by an incremented delay time t after which the NV spin state was inspected by a second laser pulse. The photons detected during the inspection pulse were then plotted as a function of time t. Two curves were recorded for each T_1 measurement, one with the NV initialized into the $m_{\mathrm{S}} = 0$ and one with the NV initialized into $m_{\mathrm{S}} = \pm 1$ by an additional non-selective microwave π pulse. The two curves were then subtracted and the differential signal fit to a simple decaying exponential, where T_1 is the exponential decay time.

From the fitted T_1 value, the spectral density of the magnetic noise evaluated at the electron's transition frequency (2.87 GHz) can be directly extracted,

$$S_{\mathrm{B}} = \frac{2}{\gamma^2 T_1} \tag{1}$$

where $\gamma = 2\pi \times 28\,\mathrm{GHzT}^{-1}$ is the electron gyromagnetic ratio[1].

Supplementary References

1. Rosskopf, T. *et al.* Investigation of surface magnetic noise by shallow spins in diamond. *Phys. Rev. Lett.* **112**, 147602 (2014).
